# The Emerging Role of the Mammalian Glycocalyx in Functional Membrane Organization and Immune System Regulation

**DOI:** 10.3389/fcell.2020.00253

**Published:** 2020-04-15

**Authors:** Leonhard Möckl

**Affiliations:** Department of Chemistry, Stanford University, Stanford, CA, United States

**Keywords:** glycocalyx, membrane organization, cancer, immune system, cancer immune therapy, immunosynapse, siglecs, KRAS

## Abstract

All cells in the human body are covered by a dense layer of sugars and the proteins and lipids to which they are attached, collectively termed the “glycocalyx.” For decades, the organization of the glycocalyx and its interplay with the cellular state have remained enigmatic. This changed in recent years. Latest research has shown that the glycocalyx is an organelle of vital significance, actively involved in and functionally relevant for various cellular processes, that can be directly targeted in therapeutic contexts. This review gives a brief introduction into glycocalyx biology and describes the specific challenges glycocalyx research faces. Then, the traditional view of the role of the glycocalyx is discussed before several recent breakthroughs in glycocalyx research are surveyed. These results exemplify a currently unfolding bigger picture about the role of the glycocalyx as a fundamental cellular agent.

## Introduction

**FIGURE 1:**
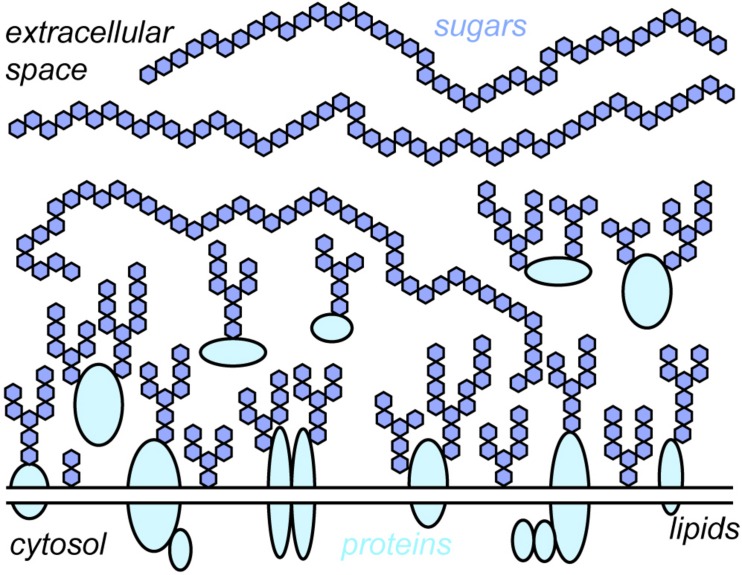
Schematic depiction of the glycocalyx. The glycocalyx is a central constituent of any cell, consisting of sugars and the proteins and lipids to which they are attached. For simplicity, the different sugars found within the glycocalyx are depicted with the same symbol (blue hexagons). Note that the depiction is roughly to scale, i.e., membrane proteins are buried under sugars.

Every cell in the human body – endothelial cells, immune cells, muscle cells, blood cells, neurons, and all the others – exhibit a glycocalyx. “Glycocalyx” literarily translates to “sweet husk.” “Sweet” indicates its key building units – various sugars (or monosaccharides) like glucose, mannose, galactose, and many others. “Husk” points toward the location of these sugars – they reside extracellularly on the cell membrane, surrounding the cell like a cloak. The sugars in the glycocalyx are connected with each other in a plethora of ways, forming sugar conjugates or “glycans.” Their sizes range from few to tens of thousands of monosaccharide units. Glycans are either free or linked to proteins, which creates glycoproteins and proteoglycans, or lipids, which creates glycolipids. The term “glycocalyx” is thus an umbrella term for the entirety of free glycans, glycoproteins, proteoglycans, and glycolipids present on the cell surface (Figure 1).

The glycocalyx has been connected to a variety of fundamental cellular and organismic events such as blastocyst implantation, embryonic development, leukocyte adhesion, or viral and bacterial infections (Matrosovich et al., 2004; Lipowsky, 2012; Weber et al., 2014; Constantinou et al., 2015; Stanley and Cummings, 2015; Formosa-Dague et al., 2018). Given the omnipresence of the glycocalyx in the human body and the importance of the processes it has been connected to, one might assume that its functional role has been investigated and understood in detail. This is, however, not the case. In contrast, the glycocalyx has been surprisingly elusive. For many decades, it was assumed that the glycocalyx merely forms a protective layer on the cell surface with no regulative potential. In retrospect, this might seem a bit surprising: The glycocalyx resides directly at the periphery of the cell and forms the interface of the cell with the extracellular space. Thus, it seems reasonable to assume that it is involved in the many processes that are related to the cell membrane.

However, there is a simple reason for the remarkable elusiveness of the glycocalyx: The glycocalyx confronts any researcher with an incredible complexity at all levels, from synthesis all the way to structural organization (see sections “From Monosaccharides to Glycopolymers” and “Biosynthesis of Glycocalyx Components”). Because of this, traditional methods to study biological systems are of very limited use when applied to the glycocalyx (see section “Problems Specific to Glycocalyx Research and Approaches to Solve Them”). Therefore, for a long time, the picture of the glycocalyx was very incomplete as tools for its detailed investigation were not available. Specifically, the prominent size of the glycocalyx – typically several 10 s to few 100 s of nanometers and thus typically burying even large membrane proteins – was underestimated for a long time.

This situation dramatically changed in recent years. Novel tools to image, model, characterize, and manipulate the glycocalyx precisely have brought it into the spotlight of biochemical and medical research. Rather than a passive coat, the glycocalyx is an active player in cell biology, chiefly involved in a range of vital cellular processes.

In this review, I will first give a brief, general introduction to fundamental concepts in glycocalyx biology. Then, I will discuss some roles that were traditionally assigned to the glycocalyx before presenting breakthrough discoveries that have revolutionized this traditional view in recent years.

## Fundamentals of Glycocalyx Biology

### From Monosaccharides to Glycopolymers

**FIGURE 2:**
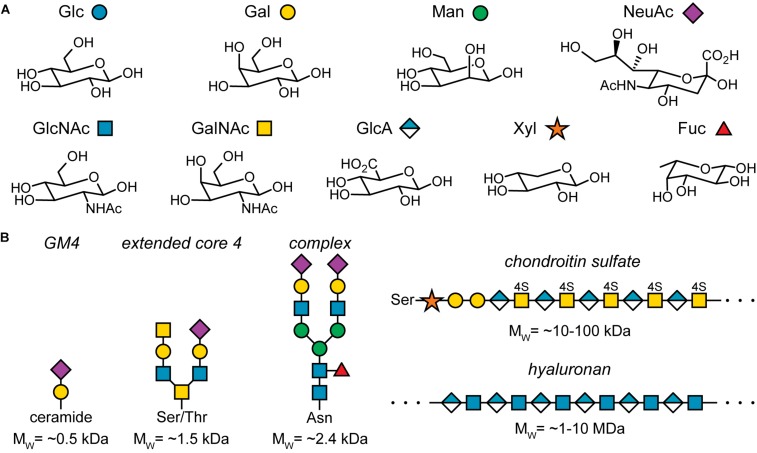
Important sugars found in humans and examples of sugar conjugates found in the glycocalyx. **(A)** Common sugars found in humans and their pictorial representation according to the symbol nomenclature for glycans (SNFG). Note that this list is not exhaustive. Glc, glucose; Gal, galactose; Man, mannose; NeuAc, *N*-acetylneuraminic acid; GlcNAc, *N*-acetylglucosamine; GalNAc, *N*-acetylgalactosamine; GlaA, glucuronic acid; Xyl, xylose; Fuc, fucose. **(B)** Five examples for sugar conjugates found in the glycocalyx: GM4, a glycolipid; an extended core 4 *O*-glycosylation structure; the core structure of complex *N*-glycosylation; a side chain found in chondroitin sulfate; hyaluronan or hyaluronic acid. For simplicity, the linkage chemistry of the glycosidic bond between each monosaccharide is not specified.

The key component of the glycocalyx are sugars or monosaccharides. They are also referred to as carbohydrates as they were initially identified as hydrates of carbon, C_x_(H_2_O)_y_. However, this name does not capture the true chemistry of sugars well. Rather, sugars can be considered polyhydroxylated carbonyl compounds. For example, glucose is an aldo-hexose, i.e., a six-membered chain of carbon atoms, where the first carbon exhibits a carbonyl group and the other five groups each carry a hydroxyl group. Fructose is a keto-hexose: Here, the second carbon atom in the chain carries the carbonyl group. Thus, the positioning of the carbonyl group and the orientation of the hydroxyl group at the chiral carbon atoms determines the monosaccharide structure. For example, glucose, galactose, and mannose are all aldo-hexoses, which differ only in the orientation of one hydroxyl group (Figure 2A, top row), emphasizing the importance of stereochemistry in glycobiology.

In solution, monosaccharides exist as a mixture between an open, chain-like form and a cyclic form. The latter is formed by an intramolecular ring closure reaction where a hydroxyl group reacts with the carbon atom carrying the carbonyl group, typically forming five- or six-membered rings with four or five carbon atoms and one oxygen atom. The ratio of open and closed form depends on the structure of the sugar, but the closed form is usually energetically more stable and thus prevalent. The carbon derived from the carbonyl group is called “anomeric carbon.” Dependent on the orientation of the hydroxyl group at the anomeric carbon, the monosaccharide exists either in the α- or β-anomer. Via ring opening and reclosing, the two anomers can interchange. For a detailed overview of the intriguing chemistry of sugars, see the literature (Lindhorst, 2007).

Figure 2A depicts the most common sugars found in the human body and their pictorial representations according to the symbol nomenclature for glycans (SNFG) (Varki et al., 2015; Neelamegham et al., 2019). As mentioned above, stereochemistry plays a central role in glycobiology. The second aspect that determines monosaccharide identity are chemical modifications such as oxidation of hydroxyl groups, *N*-acetylation, sulfation, and many more. The resulting complexity already at the level of individual monosaccharides is remarkable.

The complexity of monosaccharides is, however, just a small hint at the complexity of oligo- and polysaccharides, i.e., glycoconjugates of several to many monosaccharide units. For example, let’s consider the formation of a disaccharide that consist of two glucose units. The glyosidic bond is formed by the anomeric carbon of the first glucose unit and any hydroxyl group of the other glucose unit. As each of the two units can exist in the α- or β-anomer, there are already 19 disaccharides consisting of two glycose units. Moving to slightly larger hexasaccharides that may include all the sugars found in the human body, the number of possible isomers is at least 1.05 × 10^12^ (Laine, 1994), compared to 4096 hexanucleotides and approximately 86 million hexapeptides. Of course, not all of these possible hexasaccharide structures are realized, but the number of distinct glycoconjugates structures in the body, ranging from small conjugates of just a few hundred Da to polymers of several MDa, is still enormous (Figure 2B) (Marino et al., 2010; Gagneux et al., 2015; Stanley and Cummings, 2015).

A common way to organize glycocalyx components into major classes is to consider whether they are attached to another biomolecule or if they are free. If a glycan is attached to a lipid anchor, the resulting conjugate is called glycolipid. If one or more glycans are attached to a protein, a further differentiation is typically made: In case the glycan portion are oligosaccharides of approximately 3 to 20 monosaccharides, the resulting conjugate is called glycoprotein. In case the attached glycans are long, polymeric chains, leading to a significant sugar content, the resulting conjugate is called proteoglycan. Finally, free glycans are usually polymers, and thus, they are called glycopolymers.

Notably, the two major classes of glycoproteins are divided according to the attachment of the glycan to the protein: If the glycan is attached via a nitrogen atom of an asparagine side chain, one speaks of *N*-glycosylation and *N*-glycans. If the glycan is attached via an oxygen atom of a serine or threonine side chain, one speaks of *O*-glycosylation and *O*-glycans.

### Biosynthesis of Glycocalyx Components

The biosynthesis of glycocalyx components occurs at many places in the cell and is a complex, interlaced process. Considering the numerous in-depth reviews devoted to this topic (Spiro, 2002; Breton et al., 2006; Moremen et al., 2012; Krasnova and Wong, 2016), I will only briefly discuss it here.

For glycoproteins and glycolipids, biosynthesis is performed in the endoplasmic reticulum (ER) and the Golgi apparatus (secretory pathway). The newly synthesized protein chain is usually inserted cotranslationally into the ER. Inside the ER, the peptide chain is folded. The glycosylation procedure of the folded protein chain is dependent on the glycosylation type: *N*-glycans are preassembled and transferred as a whole onto the peptide chain inside the ER. During maturation in the Golgi apparatus, they can be further modified. For *O*-glycosylation, only a single sugar is attached in the ER, and further sugars are added stepwise in the Golgi apparatus. Glycans on glycolipids are stepwise assembled in the Golgi apparatus as well. After the synthesis is finished, the final products are packaged into secretory granules, transported to the cell membrane, and secreted.

Some glycocalyx components are not synthesized via the ER-Golgi apparatus pathway. For example, the assembly of hyaluronic acid takes place directly at the membrane by integral membrane proteins, hyaluronan synthases. Activated sugars are added to the glycopolymer chain while it is secreted into the extracellular space (Toole, 2004; Weigel and Deangelis, 2007).

The many enzymes that take part in glycan synthesis within the ER and Golgi apparatus are positioned within the secretory pathway according to the reaction they catalyze. It is, however, worth noting that many of these enzymes have a broad substrate and reaction spectrum and that they are constantly shuttled back and forth within the secretory pathway (Bieberich, 2014). Moreover, the availability of donor molecules for glycosylations, usually activated monosaccharides, strongly depend on the cellular metabolism (Lau et al., 2007). As the metabolic state of the cell is altered in cancer, this link between the cellular metabolism and the glycocalyx state directly hints at remodeling of the glycocalyx upon oncogenic events (Pavlova and Thompson, 2016; Deberardinis and Chandel, 2020). Indeed, many relations between tumor metabolism, the glycocalyx, and the cellular state have been uncovered, for example the key role of the mucin Muc1 in hypoxia survival (for a discussion of mucins, see section “The Glycocalyx Controls Cell Morphology”) (Yin et al., 2007; Chaika et al., 2012). These discoveries may be the first steps toward a unified concept linking tumor metabolism and glycocalyx state.

Taken together, glycan synthesis is regulated, but exhibits significant stochastic components, which contribute to the observed structural diversity of the synthesized glycoconjugates (Müller et al., 2016; Varki, 2017). The totality of glycosylated species of an organism or even of a single cell, the “glycome,” is thus of staggering complexity.

### Problems Specific to Glycocalyx Research and Approaches to Solve Them

As a result of the complexity of the glycocalyx at all levels, its investigation is not trivial. Many unique problems that need to be solved in order to study the glycocalyx can be named, however, three issues form the basis of the challenge:

• The glycocalyx exhibits lateral dimensions of typically several 10 s to few 100 s of nm. Its size is therefore below or close to the diffraction limit of light, which is at approximately 250 nm. Thus, the dimension of the glycocalyx can only rarely be determined with conventional optical microscopy, and fine structures are not accessible. The second major microscopy method used to study biological systems, electron microscopy, necessitates elaborate sample preparation, and reports in literature are conflicting if and how the required procedures affect the result (Ebong et al., 2011; Hegermann et al., 2016; Chevalier et al., 2017). Moreover, electron microscopy typically provides no information on the molecular identity of the imaged species. Considering the huge number of tightly packed glycans and glycoconjugates found within the glycocalyx, this is particularly unfortunate.
• The components of the glycocalyx are secondary gene products. Thus, their structure is not directly encoded in the genome, rather, it is a product of many interdependent biosynthetic pathways and furthermore subject to the metabolic state of the cell (Lau et al., 2007). Also, the enzymes involved frequently exhibit overlapping specificities. Consequently, classical genetic methods to label the glycocalyx or to identify regulatory networks during synthesis via knockouts are largely unusable.
• The difference between two monosaccharides can be as subtle as the orientation of a single functional group, but the chemical and biological effect is often tremendous. This drastically impedes specific labeling of the glycocalyx as any labeling approach must be able to precisely distinguish two glycoconjugates that are structurally almost identical.

In addition, the design of model systems, which has proven to be very beneficial for DNA and protein research, is much more challenging for glycoconjugates. Due to the intricate stereochemistry and the large number of functional groups, the synthesis of glycoconjugates is not trivial. However, pioneering work by the labs of Peter Seeberger, Geert-Jan Boons, Chi-Huey Wong, and many others, has significantly simplified this problem, and many glycoconjugates can be synthesized automatically nowadays (Schmaltz et al., 2011; Wang et al., 2013; Hahm et al., 2017; Budhadev et al., 2019; Guberman and Seeberger, 2019).

The three key challenges of glycocalyx biology fundamentally still persist, however, many inroads toward at least partially addressing them have been found. For example, if the structure of glycoconjugates in the glycocalyx are more of interest than their specific localization on the cell membrane, mass spectrometry is an excellent tool to address such questions (Pan et al., 2011; Han and Costello, 2013).

Several strategies exist to label glycocalyx components. Naturally occurring glycan-binding proteins, called lectins, have been used successfully to address individual glycan structures for various applications, however, they can suffer from low affinities or low specificities (Rudiger and Gabius, 2001; Lehmann et al., 2006; Smith and Cummings, 2013; Whelan and Bell, 2015). The creation of antibodies against glycan structures has been challenging as purification of the target glycoconjugates from a complex biological mixture at high yields is demanding. However, with recent advances in glycan synthesis (see above), it can be expected that glycan antibodies become more easily accessible (Paschinger et al., 2005; Broecker et al., 2015).

A unique approach to label specific glycocalyx components is called metabolic labeling. In this approach, cells or whole organisms are supplied with a precursor molecule, which is internalized by the cell(s) and used for glycocalyx synthesis (Keppler et al., 1995; Laughlin and Bertozzi, 2007; Baskin et al., 2010). Critically, the precursor molecule exhibits an unnatural modification, which is attached in such a way that the precursor is processed normally by the cellular biosynthetic machinery: The modification is “invisible” to the cell. Thus, the modified precursor is inserted into glycocalyx components, and the unnatural modification can be chemically addressed to attach, for example, fluorophores. Due to the bioorthogonal nature of the employed unnatural modifications, e.g., azido groups, this targeting is compatible with the requirements of living cells and organisms. This approach provides excellent specificity and high degrees of labeling and is thus very powerful. Its main drawback is that it currently only allows for targeting of individual sugars such as sialic acids or GalNAc, not reporting on the glycan structure the labeled sugar is part of.

Visualization of the glycocalyx with fluorescence microscopy is desirable as it enables to make use of the currently available and future specific labeling chemistry. This allows to assess the organization of glycocalyx components with known molecular identity. However, conventional optical microscopy is largely inapt to provide detailed insights into glycocalyx structure and dimensions due to the small size of the glycocalyx. One solution to this problem is to use super-resolution microscopy, which allows for resolutions of 10–20 nm (Moerner, 2012; Von Diezmann et al., 2017). Recently, this method has been used in combination with metabolic labeling or lectin-based staining to successfully visualize and investigate the glycocalyx in cultured cells (Letschert et al., 2014; Jiang et al., 2015; Chen et al., 2016; Möckl et al., 2019). In general, single-molecule methods seem to be valuable tools to address questions in glycocalyx research due to their unprecedented resolution and specificity (Lakshminarayanan et al., 2018).

The three key challenges of glycocalyx biology mentioned at the beginning of this section were roadblocks for a long time and prevented the appreciation of the glycocalyx as a vital cellular component. Significant process has been made over the last years to address them, but even though our understanding of the glycocalyx has improved considerably, these issues are not resolved, and further work is required.

## Functions Traditionally Attributed to the Glycocalyx

Even though the functional role of the glycocalyx for cell and membrane biology was underestimated for a long time, it would be incorrect to assume that the glycocalyx was considered to have no relevant biological purpose at all. Several functions were attributed to the glycocalyx for decades. Special attention has been paid to the endothelial glycocalyx, which lines blood vessels, in medical contexts.

The first identified function of the glycocalyx was probably protection. The glycocalyx is a dense, gel-like meshwork that surrounds the cell, constituting a physical barrier for any object to enter the cell. For example, the glycocalyx was identified to play an important role to prevent the entry of pathogens into the cell. Consequently, bacteria and viruses employ tailored mechanisms to infect (Bomsel and Alfsen, 2003; Mcguckin et al., 2011). Similarly, in the context of nanomedicine, it has been shown that the endothelial glycocalyx acts as a barrier for nanoparticle entry (Gromnicova et al., 2016; Möckl et al., 2017; Uhl et al., 2017). Also, undesired leukocyte adhesion is prevented by the endothelial glycocalyx (Lipowsky, 2012).

The endothelial glycocalyx is continuously in contact with the blood stream and acts as a vital mechanosensor on endothelial cells. Specifically, long proteoglycans with strong glycosylation such as heparan sulfate or chondroitin sulfate are involved in this process. Often, the image of wind brushing through trees is used: In this analogy, the “wind” of the blood stream acts on the proteoglycan “trees” of the glycocalyx, bending them, which creates a torque that is transferred to the inside of the cells. This leads to various intracellular responses such as release of the vasodilator nitric oxide, actin cytoskeleton rearrangement, and cell polarization (Florian et al., 2003; Thi et al., 2004; Pahakis et al., 2007; Zeng et al., 2018). Notably, interruption of the blood flow due to, e.g., ischemia/reperfusion, both pathological or due to surgery, causes substantial shedding of the endothelial glycocalyx, which leads to severe adverse effects (Rehm et al., 2007; Annecke et al., 2011).

Regarding glycocalyx regulation and organization, galectins and the galectin lattice were identified early as important agents. Galectins are a class of proteins that bind to β-galactosides, e.g., the abundant glycocalyx disaccharide *N*-acetyllactosamine (Johannes et al., 2018). Galectins exhibit either two binding sites for β-galactosides or self-assemble. Thus, they can bind multiple glycans, interconnecting them and the proteins to which they are attached. This leads to clustering of the bound glycocalyx components and formation of the so called “galectin lattice.” Consequently, receptor internalization and diffusion change, causing alterations in cellular signaling (Nieminen et al., 2007; Möckl et al., 2015), which can be also artificially induced to precisely tune the strength of lattice formation (Möckl et al., 2016).

However, it seems like significant parts of the extraordinarily complex galectin story are yet to be discovered. For example, it was recently shown that galectin-1 is shuttled to the nucleus, where it is part of morphogenesis regulation (Bhat et al., 2016). Galectin-3 has been implicated in such diverse cellular processes as organization of the primary cilium, apoptosis attenuation, and endocytosis (Furtak et al., 2001; Koch et al., 2010; Harazono et al., 2014). Interestingly, apoptosis attenuation requires heterodimerization with Bax, a protein that exhibits intriguing allostery (Jiang et al., 2016). Moreover, galectin-3 interacts with the master regulator KRAS, which, if mutated, is one of the main driver of various cancer types (Shalom-Feuerstein et al., 2008). Finally, lectins are also employed by highly pathogenic bacteria such as *Mycobacterium tuberculosis*, *Pseudomonas aeruginosa*, and uropathogenic *Escherichia coli* to facilitate cell adhesion and entry (Mitchell et al., 2002; Imberty et al., 2008; Hartmann and Lindhorst, 2011; Kolbe et al., 2019).

Considering that galectins are both glycocalyx organizing proteins and involved in a variety of cellular processes, it appears as if the glycocalyx can act as “storage compartment” for galectins and potentially other proteins. Upon triggering events, they are released and translocated into the cell, where they fulfill their respective function. This glycocalyx-controlled axis of cellular organization has likely significant impact on the state of the cell, but it is currently not understood.

## The New View of the Glycocalyx

### The Glycocalyx Controls Cell Morphology

Cell membranes can adopt a variety of morphologies. Specifically, tubular extensions have been known for decades (Kolata, 1975). Initially, it was suspected that their main role is to increase the cell-surface area, e.g., for secretion and absorption. More recent investigations showed, however, that these protrusions or membrane tubules contribute to various processes. They are relevant in such diverse areas as antigen surveillance (Jung et al., 2016), tissue development (Bischoff et al., 2013), cell signaling (Ramirez-Weber and Kornberg, 1999; Rustom et al., 2004), and vesicle formation during cancer progression (Al-Nedawi et al., 2008; Antonyak et al., 2011; Becker et al., 2016).

Intriguingly, the mechanisms that are responsible for the formation of membrane tubules were poorly understood until recently. It was hypothesized that cytoskeletal filaments push out these protrusions (Tricarico et al., 2017), but the detailed processes were not clear. One reason for this can be traced back to the challenge of creating model glycocalyces of various dimensions with exact genetic control.

**FIGURE 3:**
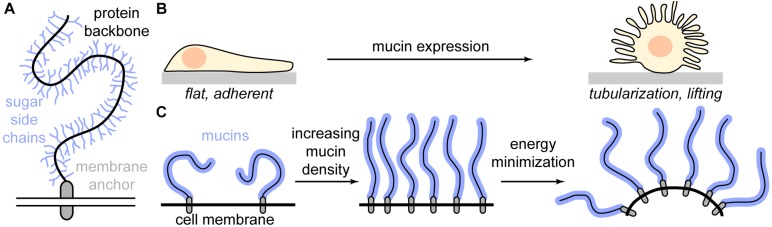
The influence of mucins on cell morphology. **(A)** Schematic depiction of a mucin. Mucins have a bottlebrush structure: A heavily *O*-glycosylated protein backbone is attached to the membrane via a membrane anchor. **(B)** Increased mucin density on the cell membrane causes a fundamental change in cell morphology from a flat, adherent phenotype to strong membrane tubularization and cell lifting. **(C)** At low densities, mucins adopt a compact mushroom phenotype. When the mucin density increases above a threshold, the mucins extend to a polymer brush. The increased order imposes an entropic penalty on the system, which is reduced via bending of the membrane, giving the mucins more orientational degrees of freedom.

Precisely this problem was addressed by a study in 2018, which introduced a method to express the mucin Muc1 with well-defined sizes in model cells (Shurer et al., 2018). Mucins are an important component of the glycocalyx and specifically relevant in the context of cancer (Figure 3A) (Kufe, 2009). They exhibit a protein backbone which is mainly composed of many tandem repeats of characteristic amino acid sequences, collectively referred to as “mucin domains.” As each domain is heavily glycosylated, whole mucins, which can exhibit persistence lengths of several microns, regularly carry more than 50% glycosylation content by mass and sometimes up to 85% (Patton et al., 1995; Felder et al., 2014; Das et al., 2015). This strong glycosylation makes mucins effectively inert to conventional proteases, severely hampering their investigation. However, a recently identified mucin-selective proteinase has overcome this limitation, which will be certainly highly beneficial for the study of mucins (Malaker et al., 2019).

The difficulty to express defined glycocalyx components is convincingly exemplified by mucins: The use of a conventional expression strategy leads to expression of mucins with a broad size distribution, typically smaller than intended, and with low yield. Using a tailored transposon-based approach, however, Shurer et al. (2019) were able to express Muc1 in model cells with high yield, precise size control, and narrow size distributions.

Building on their foundational earlier work, the group of Matt Paszek went on to study the effect of the glycocalyx on membrane morphology (Shurer et al., 2019). Strikingly, they observed that MCF10A cells, which show almost no membrane tubules when untreated, undergo massive tubularization of the membrane when mucins are expressed (Figure 3B). This effect was specific to the glycocalyx, as expression of a membrane protein without glycosylation did not induce tubularization. Considering that cancer cells frequently overexpress mucins and usually exhibit a depolarized, migratory, and tubulated phenotype, the results by Shurer et al. (2019) also provide highly relevant insights into the relationship of membrane biophysics and cancer progression.

To understand the physical mechanisms behind the tubularization of the membrane, Shurer et al. (2019) conducted a detailed theoretical analysis using a polymer brush model of the glycocalyx. Upon increasing mucin concentration in the membrane, the coiled, rather compact mucins start to interact. To avoid each other, they stretch out, forming a polymer brush (“mushroom-to-brush transition”). Bending the membrane reduces the energy of the system as each mucin gains more conformational freedom, increasing spontaneous curvature (Figure 3C). Strikingly, Shurer et al. (2019) determined the energy required to “push out” a membrane tube in the brush regime is maximally a few pN and decreases well below 1 pN for high mucin densities. At the same time, the cytoplasmic pressure required to maintain a spherical membrane bleb sharply increases and becomes quickly unphysiological. Since a single polymerizing actin filament exerts approximately 1 pN of force, the theoretical analysis explains why at a certain mucin density, membrane tubularization is the only relevant cellular phenotype.

Importantly, Shurer et al. (2019) showed that this effect is not limited to mucins. Using live synovial tissue extracted from equine carpus, they demonstrated that the same phenotype can be observed in synoviocytes. Synoviocytes are specialized cells responsible for hyaluronic acid synthesis. They exhibit a strongly tubularized membrane, very similar to the genetically modified mucin expressing cells. As treatment with hyaluronidase, degrading hyaluronic acid, completely abolished membrane tubularization, Shurer et al. (2019) verified that membrane tubularization is not specific to mucin expression, but can equally be introduced by other polymeric glycocalyx components.

It should be emphasized that this regulation of cell membrane morphology is an excellent example for an important motive: The glycocalyx frequently (although not exclusively) acts via physical interactions. Steric effects, molecular crowding, electrostatic effects (e.g., repulsion of sulfated sugars and counter ion trapping), multivalency effects, size exclusion, and others are phenomena which play a key role in glycocalyx biology (Kuo et al., 2018; Gandhi et al., 2019). Characteristically, each instance of the listed phenomena exerts only a small force on the system, but due to the large dimensions of the glycocalyx, many small forces add up to a significant total force, having a considerable effect on the cellular state.

### The Glycocalyx Is Involved in the Regulation of Membrane Protein Diffusion

Diffusion of membrane proteins, its regulation, and its interplay with membrane composition is one of the most important aspects of membrane biology (Jacobson et al., 1987, 2019; Pluhackova and Böckmann, 2015), and numerous models have been developed to describe this key process (Singer and Nicolson, 1972; Henderson and Unwin, 1975; Robertson, 1981). One model specifically addresses the interplay between membrane proteins and the cytoskeleton: The picket-fence model (Kusumi and Sako, 1996). This model proposes that “pickets,” i.e., abundant membrane proteins, bind the “fence,” i.e., the cytoskeleton close to the cell membrane, which establishes membrane domains that, for example, affect membrane protein diffusion.

In a recent study from the lab of Sergio Grinstein, an excellent candidate for a picket was identified, and the relevance of the glycocalyx was impressively shown (Freeman et al., 2018). Freeman et al. (2018) focused on phagocytosis regulation in macrophages. They showed that pickets and fences mediate receptor diffusion, functionally changing phagocytosis at a subcellular level.

**FIGURE 4:**
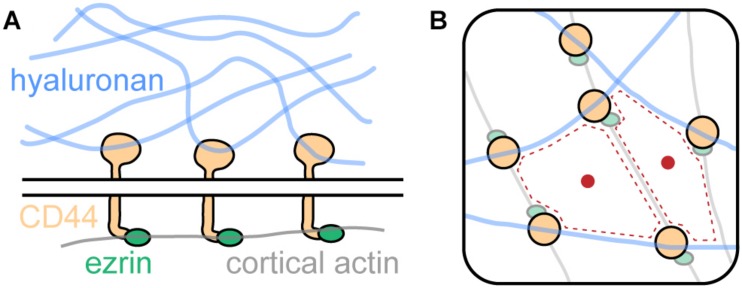
The picket-fence model and its influence onto membrane protein diffusion. **(A)** CD44, an abundant transmembrane protein, binds the glycocalyx component hyaluronic acid with its extracellular domain. Intracellularly, it engages with the cortical actin cytoskeleton via ezrin and other linker proteins. **(B)** Hyaluronan, CD44, and cortical actin demarcate membrane domains (red dotted line) that regulate diffusion of membrane proteins (red dot).

In their study, Freeman et al. (2018) identified CD44 as a bona fide picket. CD44 is highly abundant in the membrane of bone-marrow-derived macrophages (∼10^6^ copies per cell) and has been described earlier to be involved in various cellular contexts (Goodison et al., 1999). As shown in Figure 4A, CD44 binds intracellularly to the cortical actin cytoskeleton via linker proteins such as ezrin and others. Notably, CD44 exhibits another binding mode: Extracellularly, it engages with the key glycocalyx component hyaluronic acid. Thus, CD44 acts as picket and the cortical actin cytoskeleton as well as hyaluronan on the extracellular side as fences. Together, the three components form a superstructure which define membrane domains, regulating the diffusion of membrane proteins (Figure 4B).

Freeman et al. (2018) showed that each component of the superstructure is required for efficient diffusion control. Knockout of CD44 significantly increased diffusivity of membrane proteins. A synthetic construct consisting of the transmembrane domain of FcR and ezrin fused together was used to investigate the role of actin binding. The construct showed significantly increased diffusivity when actin binding of ezrin was disrupted via a point mutation in the ezrin domain. Finally, overexpression of hyaluronic acid synthase 3 greatly reduced membrane protein diffusion. Thus, the three components of the picket-fence structure (CD44, hyaluronan, and cortical actin) act together in regulation of membrane protein diffusion.

Importantly, the regulation of membrane protein diffusivity has a substantial effect on subcellular organization and phagocytosis by the studied macrophages. Freeman et al. (2018) found that migrating macrophages break down the CD44-hyaluronan-actin superstructure at the leading edge of the cell, but not at the trailing end. This causes higher receptor mobility at the leading edge, allowing for receptor clustering and initiation of phagocytosis in the area of the macrophage that is closest to, for example, a pathogen that needs to be engulfed.

In addition, this study also suggests a regulative potential of membrane proteins back on the glycocalyx: The interaction of a single CD44 molecule with hyaluronan is weak, but due to the huge number of CD44-hyaluronan binding events, the long hyaluronan molecules are firmly engaged. This was verified by investigating the interaction between *Salmonella typhimurium* bacteria with either wild-type or CD44-deficient macrophages. Immobile bacteria bound much more readily to CD44-deficient macrophages than to wild-type macrophages, suggesting that absence of CD44 causes a reduction of hyaluronan on the cell surfaces. Fascinatingly, motile bacteria bound equally well to both wild-type and CD44-deficinet macrophages. This indicates that the force generated by the bacterium is sufficient to penetrate the glycocalyx, which was corroborated by centrifugation experiments using opsonized beads: CD44-deficinet macrophages would bind much more beads than wild-type macrophages when no force was applied, but both macrophages types would bind similar amounts of beads when the beads were pressed onto the cell surface via centrifugation.

### The Glycocalyx Is Functionally Relevant for Cancer Development at All Stages

Over decades, evidence has accumulated that point toward a key role of the glycocalyx in cancer development and progression (Ohtsubo and Marth, 2006; Pinho and Reis, 2015; Tarbell and Cancel, 2016). For example, rewiring of the cellular metabolism via the Warburg effect characteristically changes glycosylation of membrane proteins (Dennis et al., 2009; Heiden et al., 2009). This changed glycosylation causes alterations in protein behavior, e.g., increased membrane residence times via prolonged engagement with the galectin lattice (Rudd et al., 1999; Lau et al., 2007). As a result, cellular signaling is shifted, ultimately modifying cellular behavior. Other studies have shown that the cancer glycocalyx acts as a mechanosensor in flow-regulated invasion (Qazi et al., 2013, 2016; Moran et al., 2019).

Tumor cells have turned against the healthy cells of the body and must therefore survive in a hostile environment. Specifically, formation of metastases is complicated: Circulating tumor cells are sought to be removed from the blood stream, and uncontrolled adhesion of circulating cells is challenging. Several recent findings have established that physical characteristics of the cancer glycocalyx play a major role in these processes, directly priming tumor cells for adhesion and prolonged survival.

**FIGURE 5:**
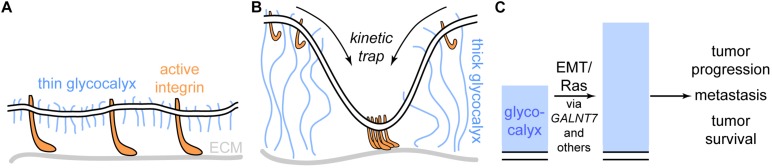
The cancer glycocalyx in integrin-mediated cell survival and its alteration upon oncogenic events. **(A)** Cells adhere to extracellular matrix (ECM) components via integrins. If the glycocalyx is thin, the integrins extend beyond the glycocalyx. **(B)** A thick glycocalyx as frequently expressed by cancer cells extends significantly further into the extracellular space than active integrins. As a result, interactions between integrins and the ECM are not possible in most areas of the cell surface, however, hotspots of integrin-ECM-binding are created via a kinetic funnel. **(C)** Upon proto-oncogenic events such as epithelial-to-mesenchymal transition (EMT) or oncogenic *RAS* activation (e.g., KRAS^G12D^), the height of the glycocalyx increases, which fosters tumor progression and survival. The oncogenic effect is mediated by mediators such as *GALNT7* and others.

Paszek et al. (2014) studied the effect of glycocalyx thickness on integrin-mediated cellular adhesion, growth, and survival. They showed that, perhaps paradoxically at first sight, a thick glycocalyx strongly increases integrin-mediated adhesion via formation of large adhesion plaques that firmly anchor the cell to the substrate, e.g., the extracellular matrix (ECM). This effect can, however, be explained via a kinetic trap model: Upon activation, integrins undergo a conformational change, causing them to extend 15–20 nm from the cell surface (Campbell and Humphries, 2011; Dai et al., 2015). Thus, a thin glycocalyx of approximately 10 nm height does not hinder the interaction of integrins with the ECM (Figure 5A). In contrast, a thick glycocalyx of several tens of nm largely prevents the interaction of integrins with the ECM. If, however, an interaction is established at some point, integrin activation will predominantly occur at the already established site of interaction. If active integrins detach from the ECM, they will likely not leave the interaction area due to the thick glycocalyx around. Thus, the thick glycocalyx primes strong integrin-mediated adhesion due to kinetic funnels or traps (Figure 5B). Strikingly, Paszek et al. (2014) found that large, bulky glycoproteins and proteoglycans such as mucins are strongly expressed on many circulating tumor cells, which emphasizes the direct clinical relevance of the revealed mechanism.

A study by Woods et al. (2017) took this idea *in vivo*. Here, mammary carcinoma cells bearing either short (∼3 nm) or long (∼90 nm) synthetic glycopolymers were injected into the tail veins of mice. The synthetic glycopolymers emulate cell-surface mucins and feature a special lipid anchor that leads to storage of the synthetic glycoproteins in intracellular vesicles. Thus, instead of degradation, the glycopolymers are recycled back to the cell surface for many days, enabling long-term studies (Woods et al., 2015). After 15 days, the mice were sacrificed and the lungs, where the injected cells migrated, were excised. The tumor burden was severely increased when the injected cells carried the long form of the synthetic glycopolymer compared to the short form. In accordance with the study by Paszek et al. (2014) and Woods et al. (2017) found that the long synthetic glycopolymer stimulated integrin-FAK mechanosensing, emphasizing the relevance of integrin-mediated tumor cell adhesion and survival.

In a recent investigation conducted by the labs of W. E. Moerner and Carolyn Bertozzi, the dimensions and nanoscale architecture of the glycocalyx was investigated with super-resolution microscopy (Möckl et al., 2019). The increased resolution allowed for direct quantification of glycocalyx dimensions from the cell membrane up. This approach was used to monitor changes in glycocalyx height upon two key oncogenic events: Epithelial-to-mesenchymal transition (EMT) and proto-oncogene activation, in this case the expression of KRAS^G12D^, one of the most prevalent oncogenic mutations known (Kalluri and Weinberg, 2009; Cox et al., 2014). They found that upon both oncogenic events, glycocalyx height significantly increased. As both EMT and KRAS^G12D^ trigger a multitude of signaling cascades, Möckl et al. (2019) went on to identify mediator genes for the effect of KRAS^G12D^ on the glycocalyx. Transcriptomic analysis revealed several promising hits, among which the galactosyltransferase *GALNT7* was most striking: In patients suffering from pancreatic adenocarcinoma, a tumor type in which *KRAS* carries an activating mutation more than 90% of the time, *GALNT7* was strongly correlated with patient survival, suggesting a relation between *KRAS* and *GALNT7*. Indeed, in KRAS^G12D^ expressing cells, siRNA-mediated knockdown of GALNT7 caused a strong reduction in glycocalyx height, indicating that *GALNT7* is indeed a single-gene mediator of the effect of oncogenic *KRAS* onto the glycocalyx. Considering the studies by Paszek et al. (2014) and Woods et al. (2017), these findings strongly suggest that cancer cells actively remodel their glycocalyx from the first oncogenic events on, which fosters tumor progression, metastasis, and survival (Figure 5C).

### The Glycocalyx Is a Key Player in Immune System Regulation and Checkpoint Inhibition

The previous sections described recent significant findings that ascertain the central role of the glycocalyx for cell morphology determination, membrane organization, and cancer progression. Each of these investigations can be readily transferred to therapeutic applications. In addition, recent studies from the lab of Carolyn Bertozzi have started to explore the potential of the glycocalyx in therapy and specifically cancer immunotherapy.

One of the hallmarks of cancer is immune evasion (Bhatia and Kumar, 2014). Normally, uncontrolled cell proliferation is recognized and terminated, but in cancer, this is no longer the case. Cancer immunotherapy therefore seeks to stimulate the immune system in order to reinstall its normal response to fight the cancer (Scott et al., 2012). This can be achieved in various ways, e.g., by using antibodies that block pathways responsible for reduced immune system activity.

The glycocalyx component that mainly constitutes the relevance of the glycocalyx for immune system regulation is sialic acid. Sialic acids are an abundant monosaccharide in the glycocalyx. Among the many cellular and organismic processes they are involved in, their role as “marker of self” is of special importance (Paulson et al., 2012; Varki and Gagneux, 2012; Blaum et al., 2015), and many studies have impressively shown the importance of sialic acids and sialic acid-binding receptors on the cell membrane, termed “siglecs” (short for sialic acid-binding immunoglobulin-type lectins, belonging to the family of I-type lectins) (Crocker et al., 2007; Jandus et al., 2014; Läubli et al., 2014). In accordance with this picture, many cancer cells overexpress sialylated proteins and lipids and their membrane, and it could be shown that this overexpression is directly involved in immune system downregulation, enabling the cancer cell to evade the attack by immune cells (Hudak et al., 2014; Boligan et al., 2015).

This strategy to dodge the immune system is very efficient. For example, one immunotherapeutic strategy to treat breast cancer is to employ a monoclonal antibody, known as Trastuzumab (Tras). Tras binds to the overexpressed cancer cell surface protein human epidermal growth factor receptor 2 (HER2) and induces an immune response which leads to internalization and downregulation of HER2 (Bange et al., 2001). Furthermore, Tras binds to FCγRIII (also known as CD16), which is expressed on natural killer (NK) cells. Thus, Tras is able to link NK cells to cancer cells, prolonging the interaction time and increasing NK cell-mediated cancer cell killing. However, the situation is more complex. 74% of breast cancer patients show HER2 expression, but only the patient group with highest HER2 levels (∼20%) respond to treatment at all (Lee et al., 2011). Even more puzzling, less than 20% of this patient group shows a response to Tras alone. Even combination therapies exhibit just 50 to 80% response (Bartsch et al., 2007).

**FIGURE 6:**
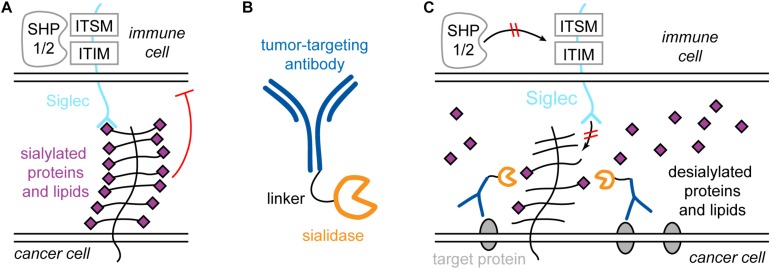
Siglecs and sialic acid in immune system regulation and specific targeting of the cancer sialome for treatment strategies. **(A)** Sialylated proteins of lipids on the cancer cell bind to siglec receptors on immune cells, e.g., natural killer (NK) cells. Siglecs contain immunoreceptor tyrosine-based inhibition motifs (ITIMs) and immunoreceptor tyrosine-based switch motifs (ITSMs), which recruit phosphatases such as Src homology 2 domain-containing protein tyrosine phosphatase 1 and 2 (SHP1 and -2), which causes activity reduction of the NK cell. **(B)** The antibody-sialidase conjugate T-Sia 2.0 to specifically desialylate cancer cells. **(C)** T-Sia 2.0 binds to membrane proteins characteristically overexpressed by the cancer. The sialidase causes desialylation of the cancer, which abolishes binding of NK cell siglecs and prevents NK cell downregulation.

Xiao et al. (2016) showed that the reason for this inefficient action of Tras is mediated by the cancer glycocalyx. It presents sialylated species to siglecs on immune cells, for example NK cells. Siglecs contain cytosolic immunoreceptor tyrosine-based inhibition motifs (ITIMs) and immunoreceptor tyrosine-based switch motifs (ITSMs). Upon binding of sialylated species on the cancer cell, SHP1 and -2 (short for Src homology 2 domain-containing protein tyrosine phosphatase 1 and 2) are recruited and activated, which causes a reduction in NK cell activity (Figure 6A). Furthermore, the binding of NK cell-activating receptors such as NK-activating receptor natural killer group 2D (NKG2D) is disrupted by hypersialylation of cancer cell glycans. Therefore, treatment with Tras may remove HER2 from the picture, but if the sialic acid- and siglec-mediated inhibition is strong enough, the patient will not show a response to Tras. Thus, sialic acids and siglecs contribute to drug resistance.

This observation directly raises the possibility to target the cancer sialome for more efficient treatment. However, broad, unspecific desialylation would lead to severe side effects due to the omnipresence of sialic acid in the organism. Thus, Xiao et al. (2016) engineered a Tras-sialidase conjugate, called T-Sia, which selectively desialylates the cancer cell. This caused enhanced activity of Tras-mediated NK cell cytotoxicity, resulting in higher potency against cells with low HER2 levels compared to treatment with Tras alone.

Recently, a smaller, improved version of T-Sia was developed by Gray et al. (2019), called T-Sia 2.0, shown in Figure 6B. The older T-Sia showed unwanted Tras-independent activity, which was caused by the affinity of the *Vibrio cholerae* derived sialidase to polyvalent substrates like cell surfaces due to the presence of lectin domains. T-Sia 2.0 employs a sialidase from *Salmonella typhimurium*, which significantly reduces Tras-independent cell desialylation. This strongly enhanced the cytotoxicity and specificity of T-Sia 2.0. Similar to T-Sia, T-Sia 2.0 binds to HER2 in the immunosynapse, where it removes sialic acids from sialylated proteins and lipids, which prevents siglecs on the immune cell from binding and restores the interaction of NK cell-activating receptors such as NKG2D with their ligands. Consequently, SHP1/2 is not recruited to the ITIM and ITSM domains of siglecs, and the immune cell activity is not downregulated (Figure 6C). Due to the improved activity and specificity of T-Sia 2.0, it was possible to study the effect of targeted cancer cell desialylation *in vivo* using a syngeneic orthotopic HER2 + breast cancer model. Treatment with T-Sia 2.0 delayed tumor growth and enhanced immune cell infiltration and activation, leading to prolonged survival. Therefore, targeted desialylation of cancer cells was established as a promising strategy to overcome glyco-immune checkpoints *in vivo*.

## Discussion and Outlook

Until recently, the glycocalyx was recognized as a cellular structure with important functions, but it was generally not assumed that the glycocalyx is an agent that is capable to fundamentally determine the cellular and organismic state. This view changed dramatically when a series of breakthrough studies over the last few years established the glycocalyx as an organelle with significant regulative potential. We now know that the glycocalyx is functionally involved at the core of cellular events of high relevance for both health and disease – from membrane organization all the way to cancer progression. Moreover, these research efforts did not halt at a mere description of the role of the glycocalyx, but also showed that the glycocalyx is an invaluable clinical target.

The glycocalyx is a fascinating melting pot of chemistry, physics, biology, and medicine. Its astounding molecular and structural complexity is deeply rooted in the diversity of carbohydrate chemistry. This complexity translates to cellular effects that touch upon various areas of cell biology: Signaling, metabolism, immunity, cell migration, adhesion, and proliferation, and many more. Finally, the complexity of the glycocalyx also frequently unites into one entity, become a force acting almost exclusively via physical forces.

Even though recent discoveries have revolutionized our view of the glycocalyx, a long way is now in front of us. New approaches need to be found to determine the detailed architecture of the glycocalyx, to understand the interplay between glycocalyx and cellular state, and to monitor its dynamic changes upon key cellular events in health and disease.

Establishing the tools required will allow for passing the first glimpses into a deeper, integrated understanding of the communication between the glycocalyx and the cell that we have acquired so far. We are yet to discover the most fascinating aspects of glycocalyx biology: The age of the glycocalyx has just began.

## Author Contributions

LM wrote the manuscript.

## Conflict of Interest

The authors declare that the research was conducted in the absence of any commercial or financial relationships that could be construed as a potential conflict of interest.
